# Feasibility study of differentiating patients with levodopa-induced dyskinesia using cerebellar gray and white matter radiomics features from 3DT1WI images

**DOI:** 10.3389/fnagi.2026.1796614

**Published:** 2026-04-22

**Authors:** Yi Chen, Yini Chen, Andong Lin, Li Ding, Linyou Wang, Zhelin Xia, Rujia Wang

**Affiliations:** 1 Department of Pharmacy, Taizhou Central Hospital (Taizhou University Hospital), Taizhou, China; 2 Department of Radiology, Taizhou Municipal Hospital, Taizhou, China; 3 Department of Neurology, Taizhou Municipal Hospital, Taizhou, China

**Keywords:** cerebellum, levodopa-induced dyskinesia, MRI, Parkinson’s disease, radiomics

## Abstract

**Background:**

Levodopa therapy effectively treats Parkinson’s disease (PD) motor symptoms but causes Levodopa-Induced Dyskinesia (LID) in some patients long-term. Cerebellar changes exist in LID cases; however, radiomics models based on this region haven’t been evaluated for diagnostic use. We built diagnostic model using cerebellar structural radiomics from 3D T1WI to non-invasively diagnose LID.

**Methods:**

In this study, we retrospectively collected 3D T1WI data from the Parkinson’s Progression Markers Initiative (PPMI) database, including data from 69 LID patients and 142 non-LID (N-LID) patients. These data were randomly split into a training set and a testing set at an 8:2 ratio. Using Fastsurfer segmentation, we identified four regions of interest (ROIs) corresponding to the left and right cerebellar gray matter and white matter. Python scripts were employed to independently extract radiomic features from each ROI. Subsequent steps involved feature selection and model construction. After selecting the optimal model, its performance was evaluated and validated. Finally, the SHAP method was used for model visualization.

**Results:**

Ultimately, the most representative 13 radiomic features were used for modeling. The model built based on the XGBoost algorithm achieved an AUC value of 0.962 on the training set and 0.849 on the testing set.

**Conclusion:**

The radiomic model extracted from the cerebellar gray and white matter effectively distinguishes between LID and N-LID patients. It offers a novel perspective on the heterogeneous characteristics of LID patients, significantly enhancing diagnostic performance and providing auxiliary support for clinical diagnosis.

## Introduction

Parkinson’s disease (PD) represents a prevalent neurodegenerative disorder among middle-aged and elderly individuals (Ben-Shlomo et al., 2024; Cramb et al., 2023; Simon et al., 2020). Its hallmark pathological characteristics encompass the degeneration and loss of dopaminergic neurons in the substantia nigra, which consequently leads to a substantial reduction in striatal dopamine levels (Chu et al., 2024). Such pathological alterations give rise to a spectrum of motor symptoms, including bradykinesia, muscle rigidity, resting tremor, and postural balance impairments (Georgiades et al., 2023; Shamim et al., 2011; Swinnen et al., 2024), accompanied by non-motor symptoms such as hyposmia, constipation, and sleep disturbances (Driver-Dunckley et al., 2014; Zheng et al., 2022), thereby exerting a profound negative impact on patients’ quality of life.

Levodopa replacement therapy stands as a pivotal intervention in the treatment regimen for PD (Dashtipour et al., 2019; Gao et al., 2016), demonstrating efficacy in alleviating motor symptoms. However, with extended duration of therapeutic administration, a subset of patients may develop LID, characterized by involuntary motor fluctuations such as end-of-dose phenomena, on-off fluctuations, and choreiform movements-symptomatic manifestations that profoundly impair patients’ quality of life (Girasole et al., 2018; Jung et al., 2021). Although the pathogenesis of LID remains incompletely elucidated, existing evidence indicates its association with dysregulation across multiple neurotransmitter systems, genetic predisposition, and environmental influences (Halje et al., 2012; Rojo-Bustamante et al., 2018; Youn et al., 2022).

A growing body of evidence underscores the significant role of the cerebellum in the pathophysiological processes associated with PD (Li et al., 2023). Beyond its involvement in motor regulation, the cerebellum is intricately linked to non-motor functions such as cognition and emotion. Nevertheless, current research predominantly concentrates on brain regions including the substantia nigra, striatum, frontal lobe, and thalamus (Jung et al., 2021; Su et al., 2023; Zhang et al., 2021). Based on findings from functional connectivity studies, there is a close link between changes in specific regions of the cerebellum and various clinical symptoms of PD. For instance, one study revealed that abnormally increased metabolic activity in the anterior lobe and vermis of the cerebellum is positively correlated with the severity of motor dysfunction; at the same time, heightened metabolism in areas I and II of the right cerebellar peduncle is closely associated with the extent of cognitive impairment (Li et al., 2023; Riou et al., 2021). This discovery provides new insights into understanding the pathological mechanisms of PD and its impact on patients’ daily lives. Notably, as a critical hub within the motor control network, structural and functional abnormalities in the cerebellum play a key role in PD-related motor impairments and may be closely connected to the pathogenesis of LID (Li et al., 2023). Anatomical evidence reveals that the cerebellum forms functional circuits with the basal ganglia through extensive fiber projections, jointly participating in motor coordination and fine motor control (Habas, 2021; Sweet et al., 2014). However, the specific alterations of the cerebellum in the development of LID in PD remain unclear and warrant further investigation.

In recent years, the diagnostic paradigm for PD has been undergoing a transformative shift from conventional clinical assessment to precision diagnosis based on biomarkers (Antoniades and Barker, 2014; Lang and Espay, 2018). For instance, Lee et al. developed a deep learning model based on baseline ^[18F]^FP-CIT PET imaging to predict whether PD patients would develop LID within 5 years after initiating levodopa treatment (Lee et al., 2025). This evolution not only facilitates early disease detection but also enables effective differentiation of clinical subtypes with distinct prognostic characteristics, thereby accelerating the development of novel disease-modifying therapies (Berg et al., 2021; Eisinger et al., 2017; Lang and Espay, 2018). Particularly, precise diagnosis of LID holds critical significance for optimizing treatment strategies and curbing disease progression in PD (Eisinger et al., 2017; Lang and Espay, 2018). Currently, clinical diagnosis of LID remains heavily reliant on subjective evaluation of symptomatic manifestations and physician expertise, necessitating urgent establishment of objective biomarkers and imaging-based assessment systems. Against this backdrop, radiomics-an emerging interdisciplinary field-leverages high-throughput quantitative feature extraction and advanced data analytics to systematically decipher latent biological information embedded within medical images, opening new avenues for disease diagnosis, prognostic stratification, and prediction of therapeutic responses (Chen et al., 2024a; Chen et al., 2025; Zwanenburg et al., 2020). The synergy between radiomics and artificial intelligence has achieved multimodal data fusion in oncology, facilitating precise differential diagnosis and prediction of treatment outcomes. To some extent, this integration realizes the integration of diagnosis and treatment, offering a replicable model for other diseases (Benfante et al., 2025). Previous studies have documented specific alterations in both the structural and functional aspects of the cerebellum among patients with PD (Jung et al., 2021), suggesting that these modifications may serve as the neural substrate for the emergence and progression of LID. Notably, radiomics technology has been validated for its capacity to sensitively detect subtle microstructural changes within MRI scans of PD patients, demonstrating superior discriminative power as an auxiliary diagnostic tool (Chen et al., 2024b). Building upon these premises, we hypothesize in this study that radiomic features derived from cerebellar MRI hold significant potential for accurately diagnosing patients afflicted with LID.

## Materials and methods

### Subject

This study was conducted in accordance with the guidelines of the Declaration of Helsinki. Patient data were obtained from the PPMI database,^1^ a global, large-scale, multi-center clinical research platform designed to systematically collect multi-dimensional data from PD patients and control subjects, including clinical characteristics, imaging data, genomic information, laboratory results, and biomarkers. PD patients were evaluated using the Movement Disorders SocietyUnified PD Rating Scale (MDS-UPDRS), which includes assessing motor complications and diagnosing dyskinesia symptoms during clinical visits. LID occurrence was defined as a score ≥ 1 on MDS-UPDRS Part IV, item 4.1 (time spent with dyskinesias), item 4.2 (functional impact of dyskinesias), or by the presence of dyskinesia identified in the clinical motor examination. If any relevant assessments indicated the presence of dyskinesia during a clinical visit, the corresponding patient was defined to have PD with LID. The enrollment and exclusion of patients were determined by neurologist based on the assessment scales.

**FIGURE 1:**
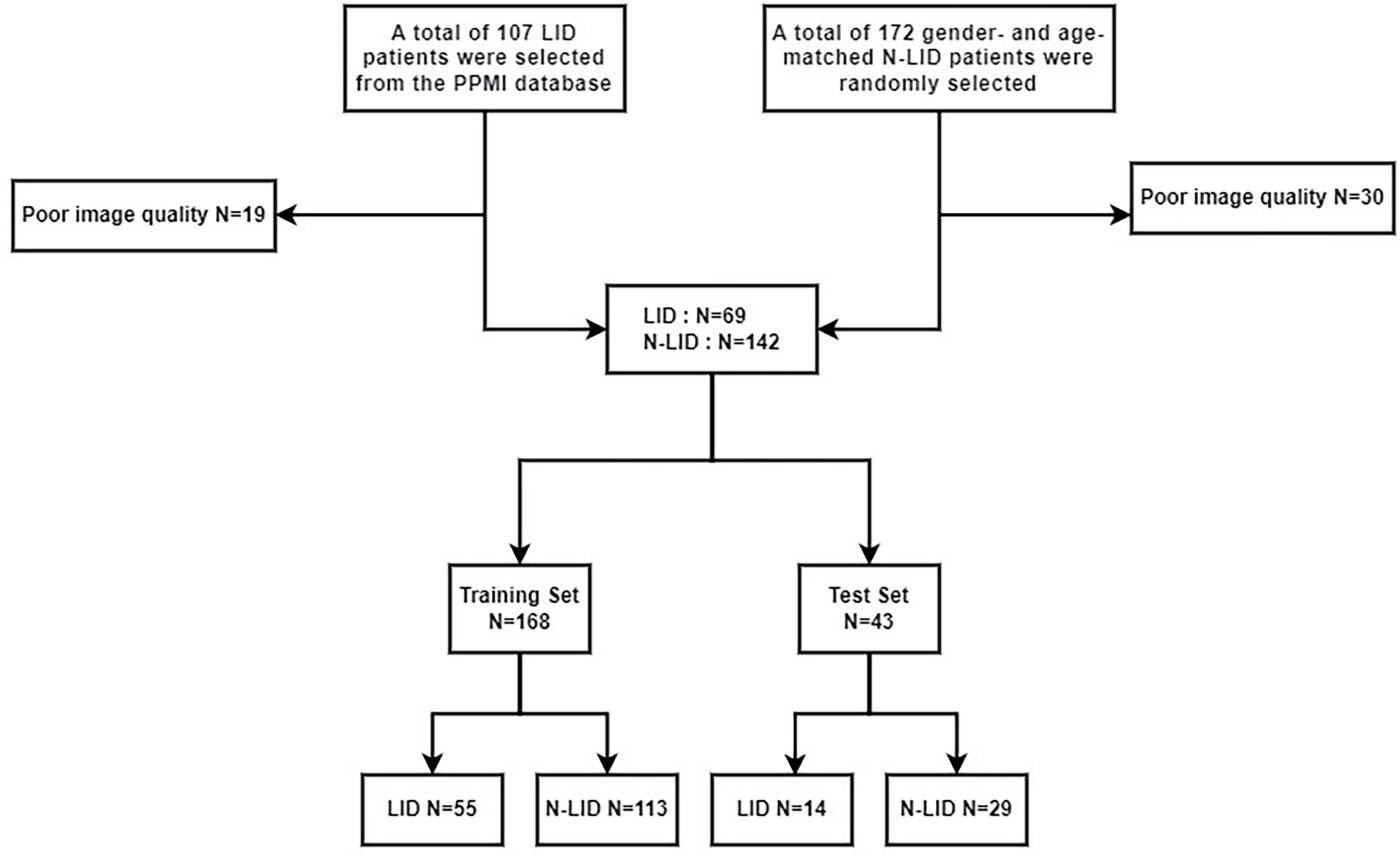
Flow diagram showing patient selection protocol and inclusion and exclusion criteria. LID, Levodopa—induced dyskinesia.

The inclusion criteria were as follows: (1) Patients diagnosed with PD according to the current diagnostic criteria of the International Movement Disorder Society; (2) Patients with LID were assigned to the LID group, while those without symptoms were assigned to the N-LID group; (3) The patients have images from the 3D T1-weighted sequence. The exclusion criteria included: (1) Poor image quality with severe artifacts or incomplete scanning. A total of 172 non-LID patients, matched to LID patients by gender and age, were randomly selected. Subsequently, we excluded patients with poor image quality (for details of the entire screening process, see Figure 1). The enrolled patients were assigned unique codes and randomly divided into a training set and a test set at an 8:2 ratio.

Each participating PPMI site received approval from an ethical standards committee on human experimentation before study initiation. Written informed consent for research was obtained from all individuals participating in the study.

### MRI acquisition

All enrolled patients were sourced from the PPMI database and underwent brain MRI examinations, including acquisition of the 3D T1WI sequence. Detailed scanning parameters are accessible via: https://ida.loni.usc.edu/pages/access/studyData.jsp?categoryId=6&subCategoryId=13.

### Image processing

#### Preprocessing

The entire image processing pipeline was implemented using Python.^2^

#### Format conversion and anonymization

Following quality control assessment, eligible DICOM datasets were converted to Neuroimaging Informatics Technology Initiative format. Concurrently, patient identifiers underwent anonymization according to standardized protocols, with enrolled subjects assigned unique coding schemas for confidentiality compliance.

### Segmentation

All participants’ 3D T1WI scans were processed using FastSurfer (Version 2.0) (Henschel et al., 2020), an open-source artificial intelligence tool designed for quantitative analysis of human brain MRI volumetry. This computational framework integrates two core modules: (1) deep learning networks enabling rapid and precise whole-brain segmentation alongside selected subcortical structures; and (2) the recon_surf utility for efficient surface reconstruction-mirroring FreeSurfer’s output capabilities while enhancing efficiency. Leveraging advanced neural architectures, FastSurfer automatically partitioned input images into 95 anatomical labels based on the DKT Atlas template.^3^ Notably, cerebellar regions were parsed into four distinct compartments: left cerebellar gray matter, left cerebellar white matter, right cerebellar gray matter, and right cerebellar white matter (Henschel et al., 2020). Segmentation examples are shown in Supplementary Figure 1. FastSurfer validated high segmentation accuracy across multiple datasets, evaluated its generalization capability, and demonstrated the robustness and precision of the segmentation.

### Radiomic feature extraction

Following image preprocessing, we employed PyRadiomics^4^ to systematically extract radiomic features from four distinct ROIs: left cerebellar white matter, left cerebellar gray matter, right cerebellar white matter, and right cerebellar gray matter. Compliance with the Image Biomarker Standardization Initiative (IBSI) guidelines (Zwanenburg et al., 2020) was strictly maintained throughout this process. For each individual ROI, a comprehensive set of 833 features was computed, encompassing: 18 first-order statistical metrics, 14 shape descriptors, 73 textural attributes, and 728 wavelet coefficients. The detailed specifications of these features are documented in Supplementary material. Consequently, a total of 3,332 radiomics features were generated across all four ROIs (833 features × 4 ROIs).

### Radiomic feature selection

This study employed a multi-step feature selection strategy to process and screen radiomic features. Firstly, the dataset was randomly partitioned into training and testing sets at an 8:2 ratio. Feature selection was performed on the training set data. Feature normalization (Z-score) and selection were performed strictly on the training set. To eliminate scale differences among features, all features underwent Z-score normalization. The feature selection process followed a three-stage pipeline: initially, hypothesis testing was conducted-features conforming to normal distribution (verified by Shapiro-Wilk test) were analyzed using independent samples *t*-tests, while non-normally distributed features were assessed via Mann-Whitney U tests, retaining those showing statistical significance (*p* < 0.05). Subsequently, Pearson correlation analysis was performed to remove redundant radiomic features with a threshold set at 0.9. Then, the top 30 features were selected based on the minimum Redundancy-Maximum Relevance (mRMR) algorithm. Finally, the least absolute shrinkage and selection operator (LASSO) method was applied for further refinement, with 10-fold cross-validation used to validate algorithm stability. These steps aimed to construct robust radiomic models and establish corresponding computational formulas.

### Model construction

**FIGURE 2:**
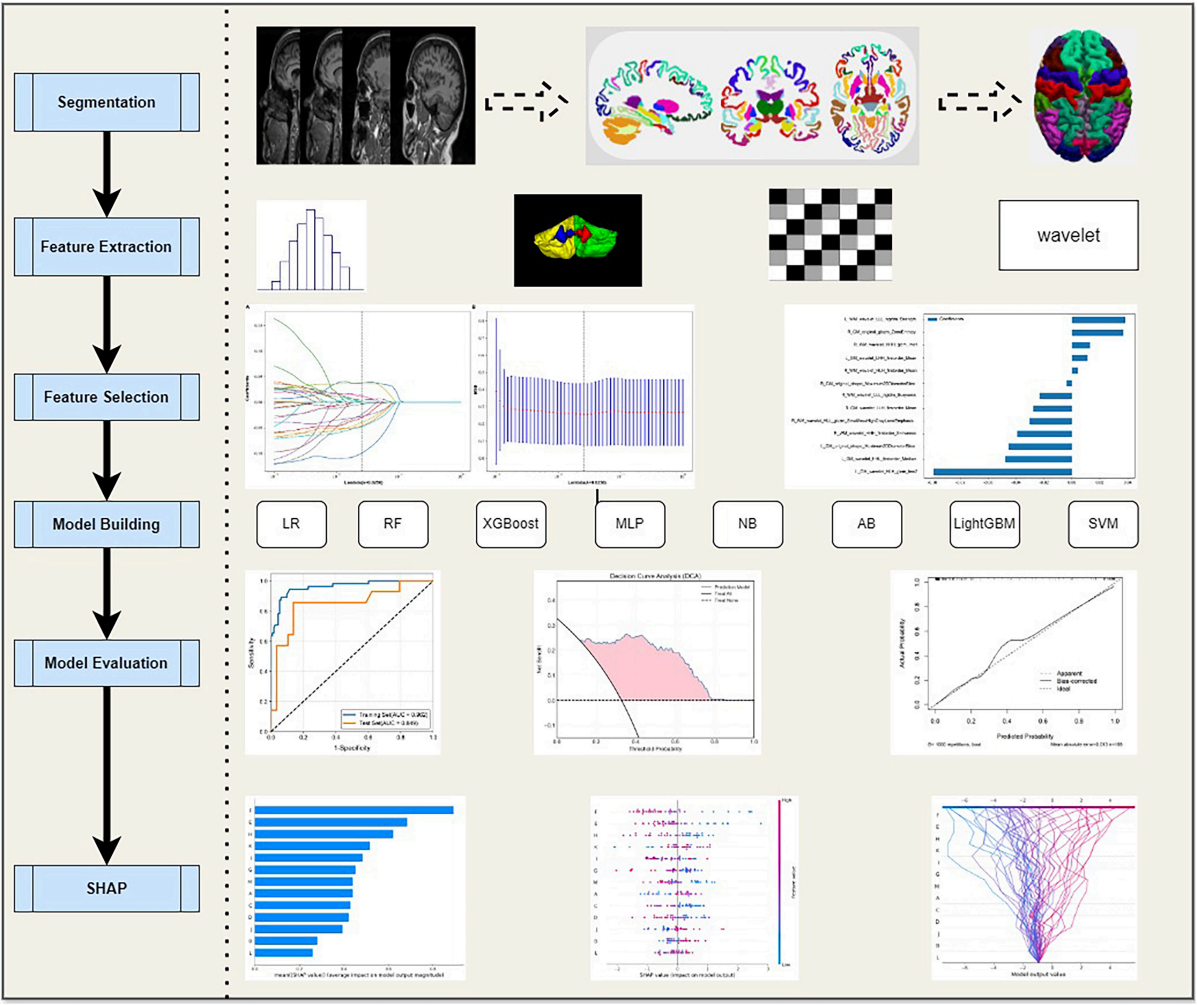
The workflow of radiomic analysis in the current study. LR, Logistic regression; RF, Random Forest; XGBoost, eXtreme Gradient Boosting; MLP, Multilayer Perceptron; NB, Naive Bayes; AB, AdaBoost; LightGBM, Light Gradient Boosting; SVM, Support Vector Machine.

After comprehensive feature selection, eight distinct algorithms were utilized to build diagnostic models: logistic regression (LR), naive Bayes (NB), Support Vector Machine (SVM), random forest (RF), Light Gradient Boosting Machine (LightGBM), extreme gradient boosting (XGBoost), AdaBoost (AB), and multi-layer perceptron (MLP). All models were trained and evaluated using Python’s scikit-learn library,^5^ implementing five-fold cross-validation for performance assessment (as detailed in Figure 2).

### Model performance evaluation

To further strengthen the rigor of validation and minimize overfitting, we maintained the five-fold cross-validation framework to systematically evaluate the robustness of model performance. The performance of each model was quantitatively assessed using a comprehensive set of metrics, including the area under the receiver operating characteristic curve (AUC), accuracy (ACC), sensitivity (Sen), specificity (Spe), F1 score, recall, positive predictive value (PPV), and negative predictive value (NPV) (as detailed in Figure 2). All analyses adopted a significance threshold of *P* < 0.05.

### SHAP visualization

SHAP (SHapley Additive exPlanations), grounded in Shapley values from game theory, enables deep interpretability by precisely quantifying the marginal effects of individual features on model predictions (Chen et al., 2024a; Chen et al., 2024b; Ma et al., 2021). Its essence lies in decomposing complex model outputs into additive contributions from input features while adhering strictly to axioms like consistency and symmetry-ensuring fair and intuitive feature importance evaluation with transparent explanatory pathways for decision-making.

### Statistical analysis

All statistical analyses were performed using R (version 4.4.2) and Python (3.9.7) packages. To compare clinical data between the two groups, appropriate statistical tests were selected based on data distribution characteristics: For normally distributed continuous variables, Student’s *t*-tests were used, with results presented as mean ± standard deviation (Mean ± SD). For non-normally distributed continuous variables, non-parametric tests (such as the Mann-Whitney U test) were employed, with results reported as median (interquartile range). Chi-square tests were used for between-group comparisons of categorical variables. A *P*-value < 0.05 was considered statistically significant. Calibration curves were plotted to assess the agreement between predicted and actual probabilities, and the Hosmer-Lemeshow test was used to evaluate model fit; a *P*-value > 0.05 indicated good model fit. Additionally, decision curve analysis (DCA) was conducted to evaluate the model’s net clinical benefit, quantifying its clinical utility across different probability thresholds.

## Results

### Demographic and clinical data of patients

A total of 211 PD patients were enrolled in this study, randomly assigned at an 8:2 ratio into a training set (*n* = 168) and a testing set (*n* = 43). Within the training cohort, there were 113N-LID cases and 55 LID cases; correspondingly, the testing cohort comprised 29 N-LID and 14 LID cases. Table 1 provides detailed demographic information and clinical characteristics across both datasets. No statistically significant differences were observed between the two groups in terms of age or gender distribution; however, a statistically significant difference was observed in Hoehn & Yahr (HY) staging, both within the training set and the testing set (*P* < 0.05).

**TABLE 1:** Demographics and clinical characteristics of the participants included in this study.

	Training set (*N* = 168)				Test set (*N* = 43)			
	N-LID (*n* = 113)	LID (*n* = 55)	Statistic	*P*	N-LID (*n* = 29)	LID (*n* = 14)	Statistic	*P*
Age	64.00 (57.00, 70.00)	63.00 (57.00, 70.00)	Z = –0.33	0.738	59.00 (54.00, 64.00)	63.00 (53.75, 68.50)	Z = –0.80	0.421
Sex, n(%)			χ^2^ = 1.08	0.299			χ^2^ = 0.11	0.739
Male	61 (53.98)	25 (45.45)			14 (48.28)	6 (42.86)		
Female	52 (46.02)	30 (54.55)			15 (51.72)	8 (57.14)		
Hoehn-Yahr	2.00 (1.00, 2.00)	2.00 (2.00, 2.00)	Z = –2.60	0.009	2.00 (2.00, 2.00)	2.00 (2.00, 2.00)	Z = –2.43	0.015

χ^2^, Chi-square test, Z, Mann-Whitney test, LID, Levodopa—induced dyskinesia.

### Radiomic feature extraction and selection

**FIGURE 3:**
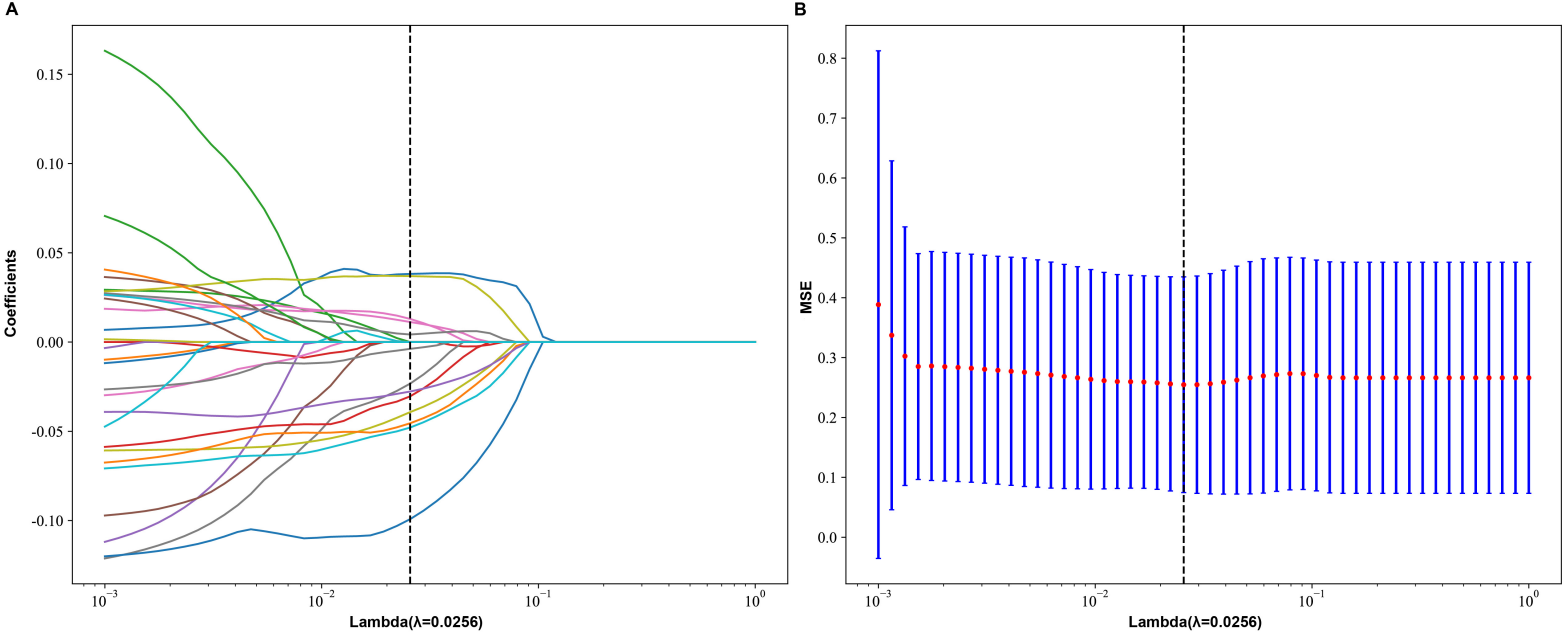
LASSO regression analysis for feature selection and model performance. **(A)** Coefficient Path Plot: This plot illustrates the behavior of the coefficients as the regularization parameter, lambda (λ), varies. Each line represents a different feature’s coefficient, showing how it shrinks toward zero as λ increases. The optimal value of λ, indicated by the dashed vertical line (λ = 0.0256), balances model complexity and diagnosis accuracy. **(B)** Mean Squared Error (MSE) Plot: This plot displays the MSE for different values of λ. The blue bars represent the MSE for each λ value, while the red dotted line indicates the average MSE across all folds of cross-validation. The optimal λ value, marked by the dashed vertical line (λ = 0.0256), corresponds to the lowest average MSE, suggesting the best trade-off between bias and variance for the model.

**FIGURE 4:**
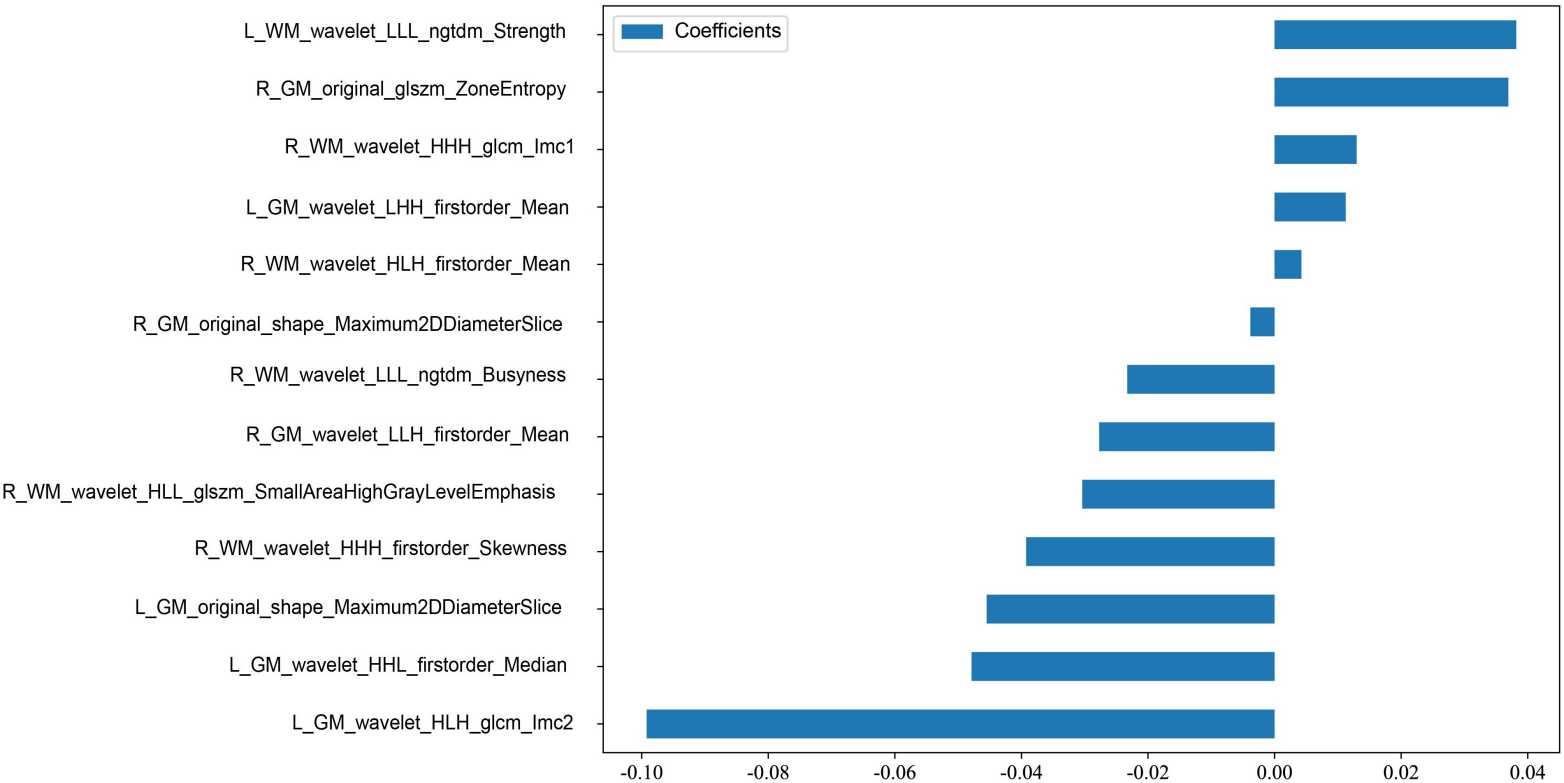
Weight distribution plot of the finally selected radiomic features.

A comprehensive pool of 3,332 radiomic features was extracted from four distinct ROIs. Employing a pre-fusion strategy, these regional features were integrated prior to selection, leveraging their collective diversity to enhance disease representation and predictive power. This strategy effectively integrates information from cerebellar gray and white matter, enabling a more comprehensive reflection of underlying heterogeneity. To standardize scale variations, all features underwent Z-score normalization before screening. Subsequent hypothesis testing identified 451 statistically significant features, which were further refined using pearson correlation thresholding (| r| < 0.9) combined with mRMR algorithm-yielding 30 representative candidates. Final dimensionality reduction via Lasso regression retained 13 optimal discriminative features. Their correlation matrix is visualized in Supplementary Figure 2, while Figure 3 illustrates the complete feature selection pipeline. The relative weights of these selected features in model prediction are displayed in Figure 4, demonstrating their respective contributions to outcome discrimination. A Rad-score, also known as a Radiomics score, uses mathematical models to combine numerous characteristics derived from radiomics into one composite indicator, aiming to streamline clinical analysis and decision-making. The Rad_score is calculated as follows:

Rad_score = 0.3274 + 0.0382 * L_WM_wavelet_LLL_ngtdm_Strength

+ 0.0112 * L_GM_wavelet_LHH_firstorder_Mean
– 0.0233 * R_WM_wavelet_LLL_ngtdm_Busyness
– 0.0392 * R_WM_wavelet_HHH_firstorder_Skewness
– 0.0479 * L_GM_wavelet_HHL_firstorder_Median
– 0.0992 * L_GM_wavelet_HLH_glcm_Imc2
– 0.0455 * L_GM_original_shape_Maximum2DDiameterSlice
– 0.0304 * R_WM_wavelet_HLL_glszm_SmallAreaHighGray LevelEmphasis
– 0.0277 * R_GM_wavelet_LLH_firstorder_Mean
+ 0.0130 *R_WM_wavelet_HHH_glcm_Imc1
+ 0.0042 * R_WM_wavelet_HLH_firstorder_Mean
+ 0.0369 *R_GM_original_glszm_ZoneEntropy
- 0.0038 * R_GM_original_shape_Maximum2DDiameterSlice.

### Model development

**FIGURE 5:**
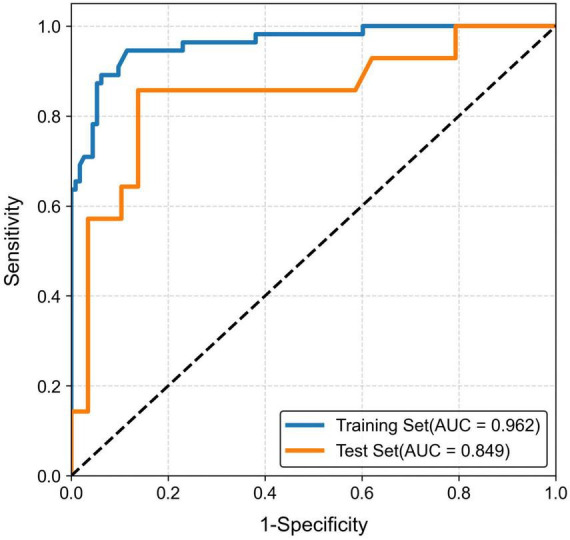
Receiver operating characteristic (ROC) curve for the selected radiomics model. Blue Line: Represents the ROC curve for the training set, with an Area Under the Curve (AUC) of 0.962. Orange Line: Represents the ROC curve for the test set, with an AUC of 0.849.

A pre-fusion strategy was implemented wherein multi-regional features were combined before applying identical modeling procedures. Eight machine learning algorithms-including Logistic Regression (LR), Naive Bayes (NB), Support Vector Machine (SVM), Random Forest (RF), XGBoost, LightGBM, AdaBoost (AB), and Multilayer Perceptron (MLP)-were utilized to construct diverse diagnostic model. The performance parameters of each model are shown in Table 2. Among these, the XGBoost algorithm emerged as optimal, demonstrating excellent goodness-of-fit on both training and testing datasets post Hosmer-Lemeshow test validation (*P* > 0.05). Performance metrics include ROC curves shown in Figure 5, DCA plotted in Supplementary Figure 3, and calibration curves presented in Supplementary Figure 3.

**TABLE 2:** Performance metrics for various machine learning models.

Model	Accuracy	AUC	95% CI	Sensitivity	Specificity	PPV	NPV	Precision	Recall	F1	Threshold	Task
LR	0.780	0.806	0.7352–0.8760	0.655	0.841	0.667	0.833	0.667	0.655	0.661	0.448	Train
LR	0.837	0.837	0.7024–0.9724	0.857	0.828	0.706	0.923	0.706	0.857	0.774	0.334	Test
NaiveBayes	0.780	0.757	0.6739–0.8395	0.600	0.867	0.687	0.817	0.687	0.600	0.641	0.675	Train
NaiveBayes	0.860	0.882	0.7738–0.9897	0.714	0.931	0.833	0.871	0.833	0.714	0.769	0.686	Test
SVM	0.744	0.719	0.6322–0.8048	0.600	0.814	0.611	0.807	0.611	0.600	0.606	0.404	Train
SVM	0.674	0.754	0.5980–0.9094	0.857	0.586	0.500	0.895	0.500	0.857	0.632	0.377	Test
RandomForest	0.762	0.891	0.8421–0.9394	0.909	0.690	0.588	0.940	0.588	0.909	0.714	0.267	Train
RandomForest	0.628	0.732	0.5819–0.8812	1.000	0.448	0.467	1.000	0.467	1.000	0.636	0.245	Test
**XGBoost**	0.905	0.962	0.9326–0.9913	0.945	0.885	0.800	0.971	0.800	0.945	0.867	0.357	Train
**XGBoost**	0.860	0.849	0.7076–0.9895	0.857	0.862	0.750	0.926	0.750	0.857	0.800	0.480	Test
LightGBM	0.714	0.674	0.5958–0.7513	0.527	0.805	0.569	0.778	0.569	0.527	0.547	0.348	Train
LightGBM	0.837	0.783	0.6425–0.9240	0.643	0.931	0.818	0.844	0.818	0.643	0.720	0.348	Test
AdaBoost	0.774	0.881	0.8315–0.9311	0.818	0.752	0.616	0.895	0.616	0.818	0.703	0.473	Train
AdaBoost	0.767	0.851	0.7300–0.9719	0.714	0.793	0.625	0.852	0.625	0.714	0.667	0.496	Test
MLP	0.804	0.767	0.6867–0.8466	0.564	0.920	0.775	0.812	0.775	0.564	0.653	0.380	Train
MLP	0.767	0.842	0.7207–0.9640	0.786	0.759	0.611	0.880	0.611	0.786	0.687	0.355	Test

ACC, Accuracy; AUC, Area Under The Curve; CI, Confidence Interval; Sen, Sensitivity; Spe, Specificity; PPV, Positive Predictive Value; NPV, Negative Predictive Value; Pre, Precision; LR, Logistic Regression; NB, Naive Bayes; SVM, Support Vector Machine; RF, Random Forest; XGBoost, eXtreme Gradient Boosting; LightGBM, Light Gradient Boosting; AB, AdaBoost; MLP, Multi-Layer Perception.

### SHAP visualization

**FIGURE 6:**
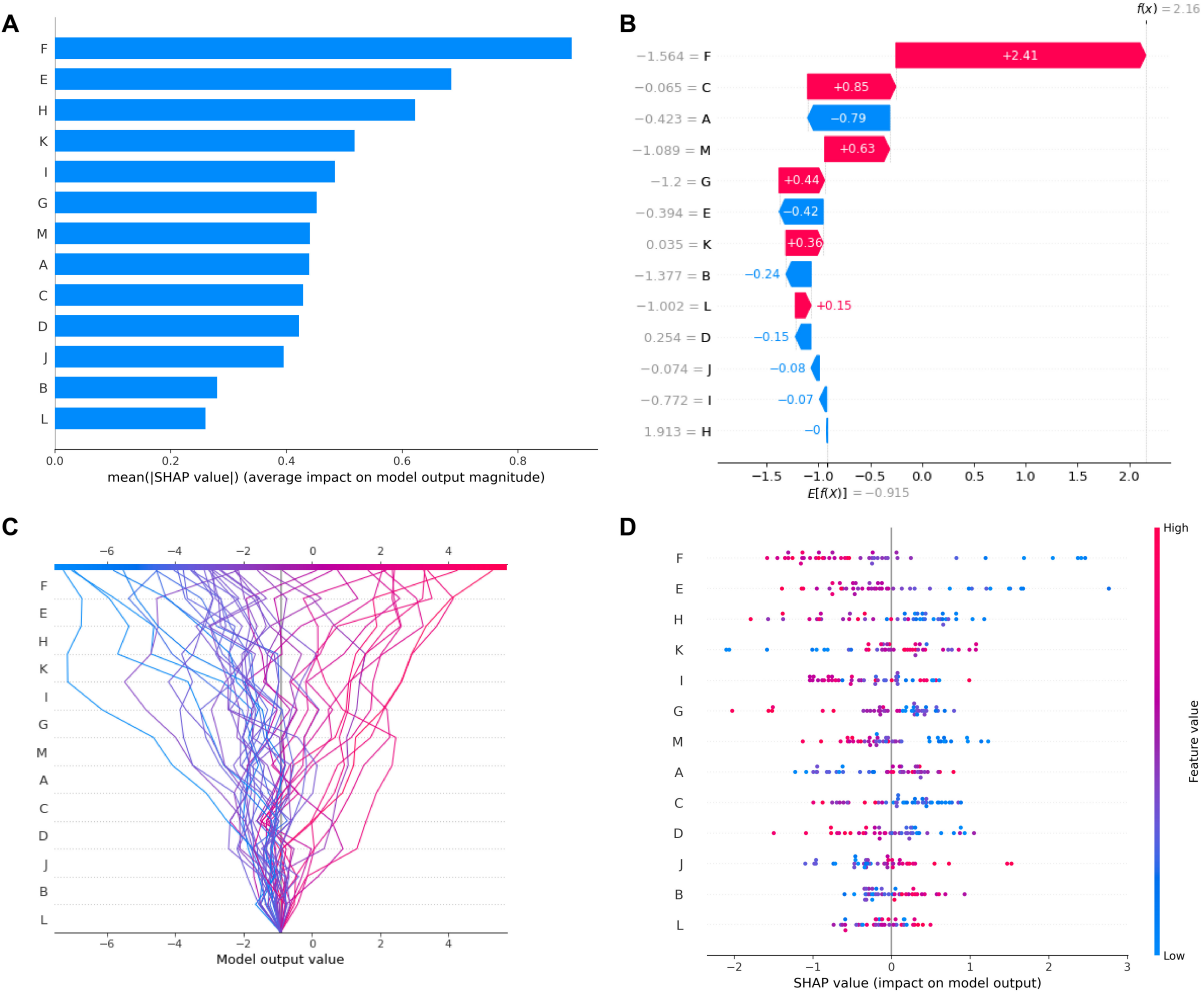
SHAP (shapley additive exPlanations) analysis of feature importance in diagnostic model. **(A)** SHAP summary plot: This plot illustrates the mean absolute SHAP values across all features, indicating their relative importance in the model’s diagnosis. **(B)** Waterfall plot of SHAP values: This chart shows the cumulative impact of features on a single predicted instance (the first sample). **(C)** Decision plot: This plot visualizes the relationship between the model output and SHAP values for each feature. The lines represent individual predictions, showing how each feature contributes to the model’s output across different instances. **(D)** Beeswarm plot: This scatter plot displays the SHAP values for each feature across all diagnosis instances. The color gradient from blue (low feature value) to red (high feature value) highlights the feature value distribution. (A: L_WM_wavelet_ LLL_ngtdm_Strength, B: L_GM_wavelet_LHH_firstorder_Mean, C: R_WM_wavelet_LLL_ngtdm_Busyness, D: R_WM_wavelet_HHH_firstorder_ Skewness, E: L_GM_wavelet_HHL_firstorder_Median, F: L_GM_wavelet_HLH_glcm_Imc2, G: L_GM_original_shape_Maximum2DDiameterSlice, H: R_WM_wavelet_HLL_glszm_SmallAreaHighGrayLevelEmphasis, I: R_GM_wavelet_LLH_firstorder_Mean, J: R_WM_wavelet_HHH_glcm_Imc1, K: R_WM_wavelet_HLH_firstorder_Mean, L: R_GM_original_glszm_ZoneEntropy, M: R_GM_original_shape_Maximum2DDiameterSlice).

To address the “black box” limitation of diagnostic model, we employed SHAP for interpretable visualization of model predictions. By quantifying each feature’s marginal contribution to output scores, SHAP values elucidate decision-making mechanisms. The selected radiomic signature model was subjected to comprehensive SHAP analysis, with detailed results visualized in Figure 6. This approach enables transparent examination of how individual imaging biomarkers collectively drive clinical predictions, enhancing both model accountability and biological plausibility assessment.

## Discussion

This study represents a pioneering effort in leveraging cerebellar gray matter radiomic features to differentiate LID in Parkinson’s disease, and we have successfully established a high-performing machine learning model. Our findings reveal that the quantitative imaging biomarkers extracted from the cerebellum can effectively distinguish between LID and non-LID patients, with the optimized model integrating 13 key radiomic signatures. Notably, this method demonstrated remarkable discriminatory capability, as reflected by AUC values of 0.962 in the training cohort and 0.849 in the test set. These outcomes not only present an innovative, non-invasive approach for the exploratory assessment of LID diagnosis but also offer substantial imaging evidence underscoring the pivotal role of cerebellar structures in LID pathophysiology. This work lays a solid foundation for future in-depth mechanism exploration and paves the way for potential clinical translation, while clearly highlighting the need for further validation before clinical implementation.

Current clinical practice primarily relies on subjective assessment of motor symptoms and medical history for LID prediction, methods plagued by suboptimal accuracy and inter-rater variability. While emerging biomarkers such as serological markers or genetic assays offer some prognostic value, their limited specificity and high implementation costs restrict widespread adoption. Although functional neuroimaging modalities including fMRI, ASL, and BOLD signals directly capture neural dynamics, they require specialized equipment, incur substantial expenses, and remain susceptible to motion artifacts (Gallichan et al., 2009; Madai et al., 2016; Mahadevan et al., 2021). In contrast, our radiomic approach leverages routinely acquired 3D T1-weighted imaging without additional sequences or contrast agents, enabling completely non-invasive evaluation. Automated segmentation algorithms coupled with standardized feature extraction protocols further minimize human bias while enhancing reproducibility-representing a significant advance toward standardized, accessible diagnostic tools for LID management.

Our findings are consistent with prior research emphasizing the cerebellum’s pivotal role in LID (Coutant et al., 2022; Jung et al., 2021; Kishore et al., 2013; Koch et al., 2009; Popa et al., 2012). Contemporary clinical and neuroimaging studies in humans have demonstrated the cerebellum’s broad engagement across cognitive, linguistic, motor, and affective domains, with growing evidence highlighting its contribution to PD pathophysiology (De Benedictis et al., 2022; Li et al., 2023). While classical frameworks attribute LID to disinhibition of cortical motor regions driven by aberrant basal ganglia output within striatal-thalamic-cortical circuits (Bezard et al., 2001; Koch et al., 2005), emerging data now support the cerebellum as a functional contributor to this process (Coutant et al., 2022; Kishore et al., 2013; Koch et al., 2009; Popa et al., 2012). Advanced imaging approaches have revealed functional specialization of cerebellar subregions—particularly the motor-related subregions encompassing lobules I–V, VI, VIIB, and VIII—which exert substantial regulatory influence on cortical motor areas (Sathyanesan et al., 2019). As a key subcortical structure involved in motor regulation and sensorimotor integration, the cerebellum is an integral component of the neural network governing movement control.

Although previous structural MRI studies did not identify significant volumetric changes in the cerebellum of LID patients, functional connectivity abnormalities have been consistently reported (Yoo et al., 2019; Zhang et al., 2022). This observation aligns with our radiomic findings, which suggest that subtle regional structural alterations may manifest as shape and texture variations detectable via radiomic analysis—capturing features that conventional volumetric assessments may overlook. The gray/white matter textural patterns we extracted are associated with established pathological processes: dopaminergic “pulse storms” in LID trigger synchronization within basal ganglia-cerebellar-cortical circuits (Kishore and Popa, 2014), leading to excitotoxic gray matter changes (e.g., dendritic-somatic shrinkage) and white matter microstructural damage (e.g., axonal/myelin disruption) (Nishijima et al., 2014; Rylander et al., 2010). These microstructural shifts are reflected in MRI signal heterogeneity, which radiomic features can quantify as changes in texture homogeneity, providing measurable indicators of LID-related neural alterations.

Collectively, our radiomic data support the hypothesis that cerebellar dysfunction is associated with the neural substrate of LID development. Notably, the radiomic features showing significant intergroup differences may reflect underlying neuronal synchronization alterations or localized microstructural reorganization. These biological changes can be indirectly represented in medical images, where differences in tissue microstructure correspond to signal intensity variations that radiomic algorithms can quantitatively capture. These findings enhance our understanding of the neural underpinnings of PD motor complications and suggest potential directions for targeted interventions. However, it is critical to emphasize that radiomic features reflect imaging-derived patterns rather than direct pathological evidence; thus, the precise molecular and cellular mechanisms linking these imaging signatures to LID require further validation through multidisciplinary studies.

### Limitations

Several limitations warrant acknowledgment in this study. First, the validation strategy of this study is currently confined to the PPMI dataset, and has not yet incorporated an independent local hold-out validation set or multicenter external validation. Future work requires further expansion of the sample size and the introduction of additional independent validation cohorts to conduct in-depth research. Second, the cohort exhibited a non-negligible class imbalance between LID and non-LID groups. This imbalance may bias model training, particularly for the minority class, and compromise the robustness of the derived radiomic features, potentially leading to suboptimal predictive accuracy for underrepresented subgroups. Third, our radiomic analysis relied solely on 3D T1-weighted imaging sequences, without comparative assessment of alternative sequences. This restriction may limit the comprehensiveness of lesion detection and the sensitivity of radiomic signatures to LID-related microstructural changes, as different sequences capture distinct tissue properties. Fourth, the regional analysis focused on four coarsely defined cerebellar regions (left/right gray/white matter), bypassing granular subregional characterization. Contemporary segmentation techniques enable precise lobule-level segmentation, which could reveal more specific associations between cerebellar subregions and LID. This coarse regionalization constrains the depth of our mechanistic insights and represents a critical avenue for future refinement. Lastly, potential confounding clinical variables (e.g., disease duration, medication dosage, motor severity scores) were not systematically adjusted for in the radiomic model. These variables may confound the association between cerebellar radiomic features and LID, introducing unquantified bias into our observed correlations. Future studies should integrate multimodal clinical data to disentangle these confounding effects.

## Conclusion

In summary, our cerebellum-derived radiomic model exhibits promising diagnostic accuracy for identifying LID patients. The extracted imaging biomarkers can effectively characterize the heterogeneous microstructural changes within cerebellar regions that are linked to LID pathophysiology. Nevertheless, before this technology can be integrated as a clinical adjunct, external validation across diverse cohorts is essential.

## Data Availability

The original contributions presented in this study are included in this article/Supplementary material, further inquiries can be directed to the corresponding authors.

## Footnotes

1
https://www.ppmi-info.org/

2
https://www.python.org/

3
https://surfer.nmr.mgh.harvard.edu/fswiki/FsTutorial/AnatomicalROI/FreeSurferColorLUT

4
https://pyradiomics.readthedocs.io/

5
https://scikit-learn.org/
