# Supplementary material for: Feasibility study of differentiating patients with levodopa-induced dyskinesia using cerebellar gray and white matter radiomics features from 3DT1WI images
Source: Front Aging Neurosci. 2026 Apr 22;18:1796614. doi: 10.3389/fnagi.2026.1796614 (PMC13143927; doi:10.3389/fnagi.2026.1796614)
Supplement: Supplementary file 1 [file Supplementary_file_1.docx]

**Supplementary Materials**

**Radiomic Feature Extraction**

config.yaml:

imageType:

Original: {}

Wavelet: {}

featureClass:

shape:

firstorder:

glcm:

- 'Autocorrelation'

- 'JointAverage'

- 'ClusterProminence'

- 'ClusterShade'

- 'ClusterTendency'

- 'Contrast'

- 'Correlation'

- 'DifferenceAverage'

- 'DifferenceEntropy'

- 'DifferenceVariance'

- 'JointEnergy'

- 'JointEntropy'

- 'Imc1'

- 'Imc2'

- 'Idm'

- 'Idmn'

- 'Id'

- 'Idn'

- 'InverseVariance'

- 'MaximumProbability'

- 'SumEntropy'

- 'SumSquares'

glrlm:

glszm:

gldm:

ngtdm:

setting:

normalize: true

normalizeScale: 100 # This allows you to use more or less the same bin width.

interpolator: 'sitkBSpline'

resampledPixelSpacing: [1, 1, 1]

Figure


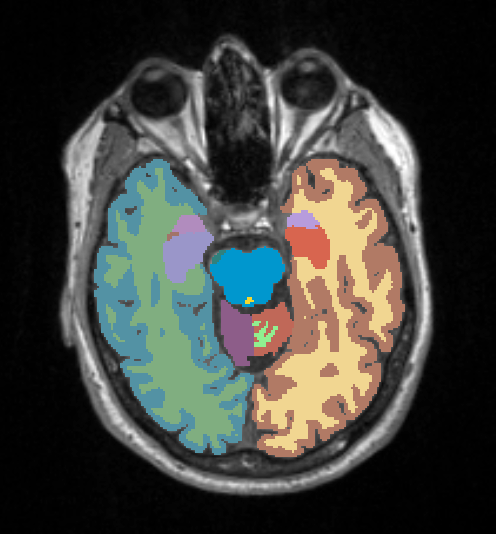


Figure S1. Segmentation Example


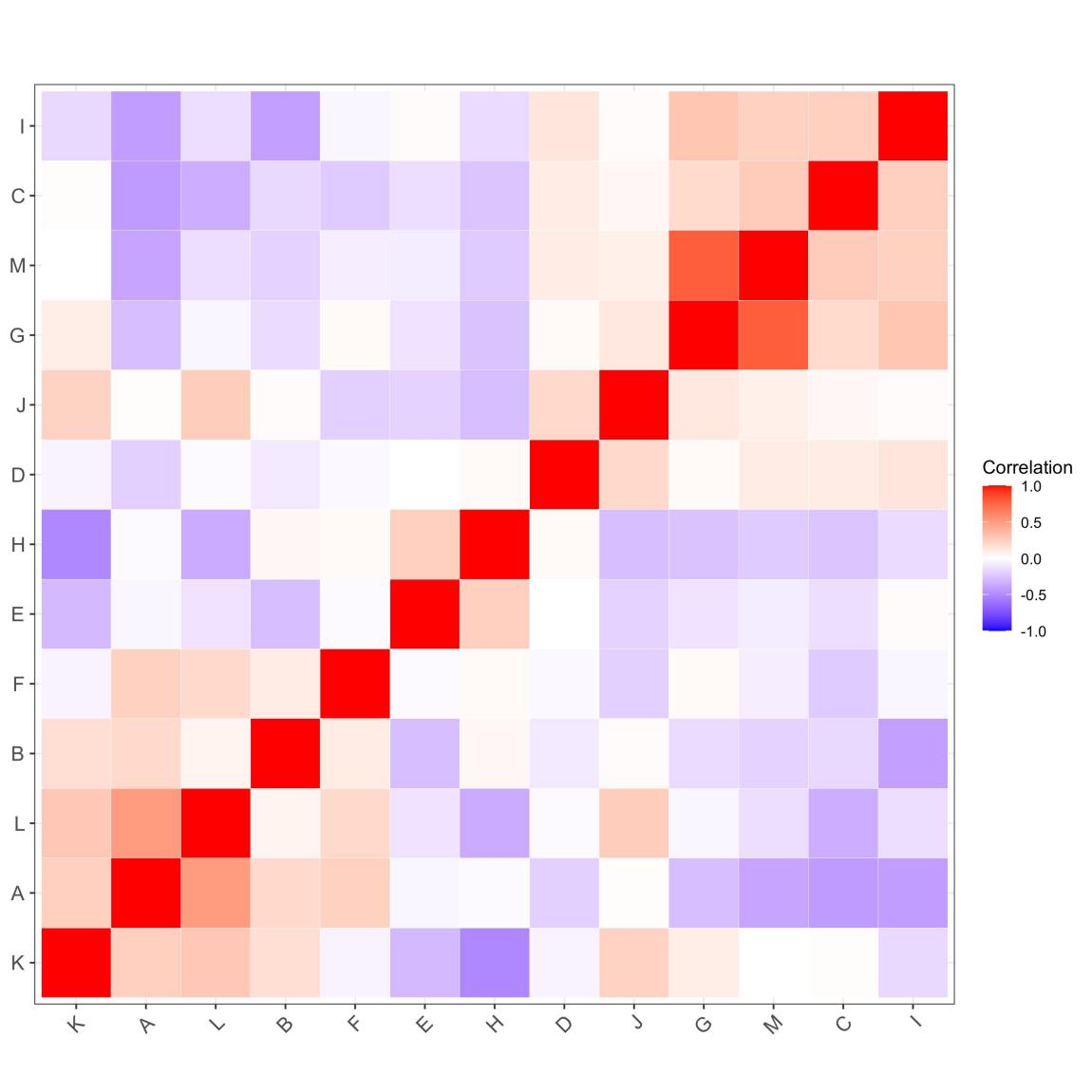


Figure S2. Heatmap representing the optimal radiomic features retained in the final model.

A: L_WM_wavelet_LLL_ngtdm_Strength, B: L_GM_wavelet_LHH_firstorder_Mean,

C: R_WM_wavelet_LLL_ngtdm_Busyness, D: R_WM_wavelet_HHH_firstorder_Skewness,

E: L_GM_wavelet_HHL_firstorder_Median, F: L_GM_wavelet_HLH_glcm_Imc2,

G: L_GM_original_shape_Maximum2DDiameterSlice, H: R_WM_wavelet_HLL_glszm_SmallAreaHighGrayLevelEmphasis,

I: R_GM_wavelet_LLH_firstorder_Mean, J: R_WM_wavelet_HHH_glcm_Imc1,

K: R_WM_wavelet_HLH_firstorder_Mean,L: R_GM_original_glszm_ZoneEntropy,

M: R_GM_original_shape_Maximum2DDiameterSlice


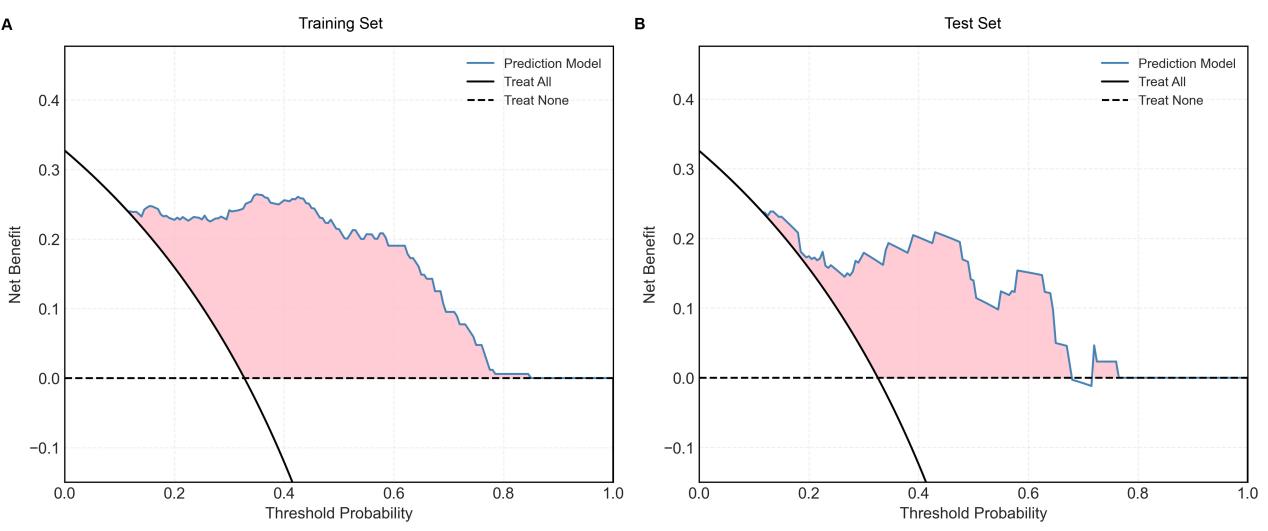


Figure S3. Decision Curve Analysis(DCA) curves of the model. A represents the training set, and B represents the test set.


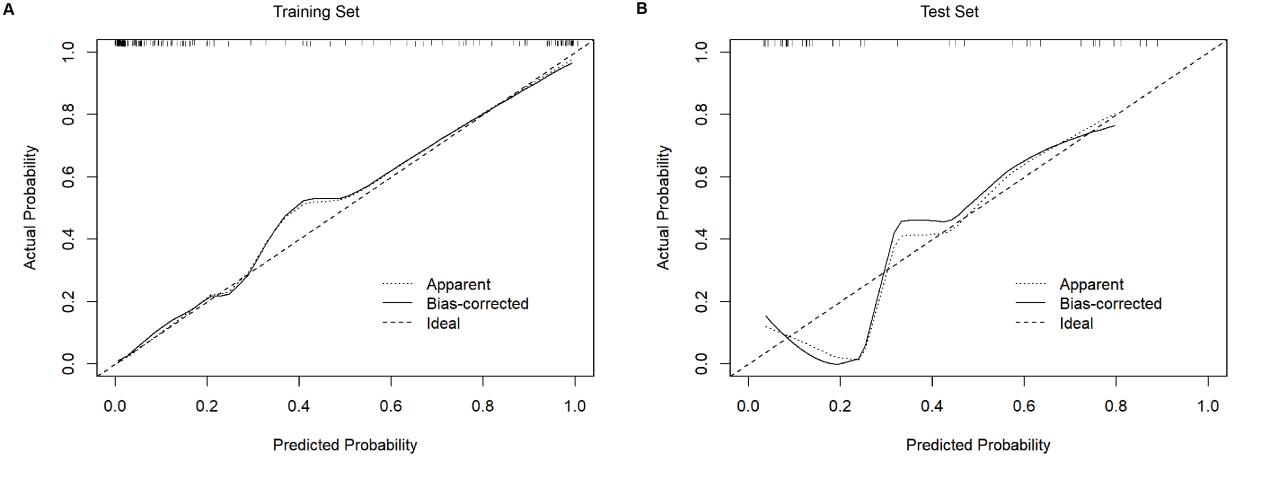


Figure S4. Calibration curves of the model. (A) Training set; (B) Test set. The model shows good fit on both datasets.
